# A Role for Peripheral Anandamide and 2-Arachidonoylglycerol in Short-Term Food Intake and Orexigenic Hypothalamic Responses in a Species with Continuous Nutrient Delivery

**DOI:** 10.3390/nu13103587

**Published:** 2021-10-13

**Authors:** Isabel van Ackern, Angela Kuhla, Björn Kuhla

**Affiliations:** 1 Research Institute for Farm Animal Biology (FBN), Institute of Nutritional Physiology ‘Oskar Kellner’, Wilhelm-Stahl-Allee 2, 18196 Dummerstorf, Germany; vanAckern@fbn-dummerstorf.de; 2 Rostock University Medical Center, Institute for Experimental Surgery, Schillingallee 69a, 18057 Rostock, Germany; angela.kuhla@uni-rostock.de

**Keywords:** endocannabinoids, food intake regulation, hypothalamus, immunohistochemistry

## Abstract

The endocannabinoid system (ECS) plays a pivotal role in the complex control and regulation of food intake. Pharmacological ECS activation could improve health in energy-deficient stages by increasing food intake, at least in intermittent feeders. However, knowledge of the mechanism regulating appetite in species with continued nutrient delivery is incomplete. The objectives of this pilot study were to investigate the effect of the intraperitoneal (i.p.) administration of the endocannabinoids (ECs) anandamide (AEA) and 2-arachidonoylglycerol (2-AG) on food intake, plasma EC concentrations and hypothalamic orexigenic signaling, and to study how the circulatory EC tone changes in response to short-term food deprivation in dairy cows, a species with continuous nutrient delivery. The administration of EC resulted in higher food intake during the first hour after treatment. Plasma AEA concentrations were significantly increased 2.5 h after AEA injection, whereas plasma 2-AG concentrations remained unchanged 2.5 h after 2-AG injection. The hypothalamic immunoreactivity of cannabinoid receptor 1, agouti-related protein, and orexin-A was not affected by either treatment; however, neuropeptide Y and agouti-related protein mRNA abundances were downregulated in the arcuate nucleus of AEA-treated animals. Short-term food deprivation increased plasma 2-AG, while plasma AEA remained unchanged. In conclusion, i.p.-administered 2-AG and AEA increase food intake in the short term, but only AEA accumulates in the circulation. However, plasma 2-AG concentrations are more responsive to food deprivation than AEA.

## 1. Introduction

The control and regulation of food intake is a complex process involving central and peripheral signals that are integrated at the brain level [1,2]. Endocannabinoids (ECs) may act at both peripheral and central sites, thus exerting a pivotal role in regulating energy homeostasis [3,4]. The endocannabinoid system (ECS) consists of the G protein-coupled cannabinoid receptors 1 (CB_1_) and 2 (CB_2_) [5], their endogen ligands N-arachidonylethanolamide (anandamide; AEA) and 2-arachidonoylglyerol (2-AG) [6,7] and specific enzymes for their synthesis and degradation [8]. In the brain, CB_1_ receptors are, among others, expressed in areas controlling food intake, such as the paraventricular nucleus (PVN) of the hypothalamus [9]. In peripheral organs, CB_1_ is predominantly expressed in the enteric nervous system of the intestine and at terminal ends of the vagus nerve [10,11], thus enabling ECs to engage in afferent communication along the gut–brain axis [12,13]. As derivatives of arachidonic acid, ECs are produced ubiquitously within the body. Bound to serum albumin [14], ECs can be distributed throughout the body via the circulation and in the case of AEA, be stored in intracellular adiposomes [15,16]. The cellular uptake and storage of AEA potentially extends the previously reported short half-life of circulatory 2-AG and AEA of only a few minutes [17,18]. AEA and 2-AG function as lipophilic signaling molecules and modulate the release of various neurotransmitters [19]. In rodents, intraperitoneally administered ECs have been shown to induce hyperphagia and increase energy intake [20,21,22] by activating vagal [12] and hypothalamic CB_1_ receptor signaling [23]. Thus, ECs partake in the control of homeostatic feed intake, whereas their action in the limbic system induces hedonic feed intake [4,24]. In the hypothalamus, ECs exert neuromodulatory effects by activating major orexigenic neurons located either in the arcuate nucleus (ARC), e.g., neuropeptide Y (NPY) and agouti-related protein (AgRP) neurons, or in the lateral hypothalamus (LH), e.g., orexin-A (OX-A) neurons [9]. The pharmacological stimulation of CB_1_ receptors augments hypothalamic NPY release [25], presumably through EC-mediated disinhibition via CB_1_-containing axons innervating AgRP/NPY neurons [26]. Furthermore, EC-mediated CB_1_ activation has been shown to enhance OX-A activity in the LH of mice [27], and a CB_1_ and OX receptor heterodimerization has been demonstrated in various cell lines [28,29,30] as well as in the LH of mice [27].

As proposed by Allen et al. [31], cows provide an ideal animal model to study the regulation of food intake in species with continuous nutrient supply at different physiological stages [32]. In addition, cows allow frequent blood sampling with almost no or minimal disruption of food intake behavior. However, so far, little is known about the cow as an animal model for endocannabinoid research and about the involvement of the ECS and the impact of the EC tone in the regulation of food intake in ruminants. In a few pioneer studies, the relationship between the expression of genes related to the ECS or plasma EC concentrations and the energy status of cows have been investigated [33,34,35]. The baseline EC tone and ECS gene expression in cows has been investigated in bovine liver [33], subcutaneous adipose tissue [34] and the hypothalamus [35]. Specifically, feeding a ration exceeding energetic requirements increased the hepatic expression of CB_2_ [33], and high postpartum weight loss and lipolysis increased the AEA and 2-AG content and expression of CB_1_ and CB_2_ in adipose tissue in cows [34]. Furthermore, Kuhla et al. [35] showed that increased plasma AEA and 2-AG concentrations postpartum are directly associated with the level of food intake in cows postpartum. However, so far, no study testing the cause–effect relationship between ECs and food intake has been performed in cows. Therefore, the objectives of this pilot study were to evaluate the effects of intraperitoneal (i.p.) AEA and 2-AG administration on systemically available EC concentrations, food intake and hypothalamic orexigenic signaling, and to further investigate how the circulatory EC tone changes in response to short-term energy deprivation.

## 2. Materials and Methods

### 2.1. Animals and Experimental Design

All animal experiments were conducted in accordance with the ARRIVE guidelines (https://arriveguidelines.org; accessed on 1 September 2018), the German Animal Welfare Act and approved by the ethical committee of the State Mecklenburg—Western Pomerania, Germany (Registration No. LALLF M-V 7221.3-1.1-041/18).

Experiment 1: Twenty non-pregnant Simmental cows from 1st to 10th lactation (>120 days in milk, DIM) with a mean body weight (BW) of 747 ± 15 kg and an average milk yield of 15.6 ± 1.1 L per day entered the experimental trial in five blocks of four. Each block consisted of a 20-day adaptation period followed by two 4-week feeding periods, each offering a different diet (Figure S1). Diets were fed in a cross over design, and the first 20 days of the second feeding period was considered the washout period. Diets were offered as total mixed ration (TMR, Table S1) and were based either on grass silage (GS) or corn silage (CS). Cows were housed in an experimental free-ranging barn at the Institute for Farm Animal Biology, Dummerstorf, Germany, and fed at 05:00 a.m. and again at 05:00 p.m. for ad libitum intake. Access to food was restricted between 05:00 a.m. and 08:00 a.m. to ensure a comparable start of food intake at 08:00 a.m. Cows were milked twice daily (05:30 a.m. and 05:30 p.m.) and milk yield from the evening and following morning milking was summarized as the daily milk yield. The BW was recorded after each milking to calculate the mean metabolic BW (BW^0.75^) as the weekly mean. After balancing for BW^0.75^, age, milk yield and DIM, animals were allocated to three groups. The two EC treatment groups were injected either with 2.5 µg 2-AG/kg BW (*n* = 6) or 5 µg AEA/kg BW (*n* = 7), purchased from Tocris (Bioscience, Bristol, UK). Doses in the low microgram/kg range were chosen because they were most effective in food intake response studies in rodents [20,22] and exert no unwanted cannabimimetic side effects [36,37]. The ECs were pre-dissolved in ethanol and diluted in 8 mL of 0.9% NaCl right before injection. The control group was injected with 8 mL of a sterile 0.9% NaCl solution (*n* = 7). Treatments were administered daily during the last 8 days of each feeding period at 08:00 a.m. via i.p. injections (1.20 mm × 80 mm, SUPRA, Vivomed, Geislingen, Germany) into the right paralumbar fossa. The i.p. route of administration was chosen to allow absorption from the peritoneal cavity into the systemic circulation, as well as to activate the gastrointestinal ECS and vagal afferents of the gut–brain axis [12,13]. From day 6 of each treatment period onwards, cows were kept in tie-stalls to prevent social interaction during food intake. Cows were fitted with a jugular vein catheter (Cavafix Certo Splittocan 338, B. Braun Melsungen AG, Melsungen, Germany) connected to a 4 m extension line to allow blood sampling without interference of intake behavior. On day 8 of each treatment period, blood samples were collected in EDTA-containing tubes 1 h before, 2.5 and 5.5 h after i.p. administrations. Cows kept in tie-stalls were milked at 06:00 a.m. and 04:00 p.m. Troughs were emptied at 06:00 a.m. and cows were given access to food at 08:00 a.m. and 05:00 p.m. Food intake was recorded as disappearance from trough every 6 min by an electronic registration device (PAARI, Erfurt, Germany). The food dry matter (DM) content was determined, and dry matter intake (DMI) was calculated. At the end of the second feeding period (day 9 of the treatment period), an additional i.p. injection was given at 07:00 a.m., approximately 1 h before slaughter of the control and approximately 2 to 3 h before slaughter of the EC-treated animals. After transfer to the institute’s slaughterhouse, located approximately 300 m from the stall, cows were anesthetized by captive bolt stunning and exsanguinated, and the hypothalamus was isolated within 20 min post mortem.

Experiment 2: Seven lactating, non-pregnant Simmental cows from 1st to 6th lactation (>120 DIM) with a mean BW of 778 ± 25 kg and an average milk yield of 16.7 ± 2.5 L per day were used to study the response of plasma EC concentrations relative to energy deprivation. The cows were kept in a free-ranging barn and fed a CS-based TMR (Table S1), as described above. For food deprivation, troughs were emptied and access to the Roughage Intake Control system (RIC, Insentec B. V., Marknesse, The Netherlands) was blocked between 07:00 a.m. and 02:00 p.m. At 02:00 p.m., troughs were refilled, and cows were given back their access to ad libitum intake. Immediately before the start of the food deprivation period, at the end of 7 h food deprivation, and 17 h after refeeding, a blood sample was taken from the tail vein and collected in EDTA-containing tubes.

### 2.2. Analyses of Plasma Endocannabinoids and Metabolites

For the analysis of plasma EC concentrations in experiment 1 and 2, blood samples were immediately placed on ice, centrifuged at 1570× *g* for 20 min at 4 °C, and stored at −80 °C. Samples were analyzed for AEA and 2-AG concentrations by the Research Core Unit Metabolomics at the Hannover Medical School using the cross-validated method as described recently [38]. Briefly, analyses were carried out using a Waters ACQUITY UPLC-MS/MS system with a tandem quadruple mass spectrometer XEVO TQ MS (Waters, Milford, MA, USA), using a Waters ACQUITY BEH C18 column (100 mm × 2.1 mm i.d., 1.7 µm particle size) for separation of analytes. Plasma-free, non-esterified fatty acid (NEFA) and glucose concentrations were analyzed spectrophotometrically at a semi-automatic analyzer (ABX Pentra 400, HORIBA Medical, Kyoto, Japan) using the respective NEFA-HR 91797 (FUJIFILM Wako Chemicals Europe GmbH, Neuss, Germany) and Glucose HK CP A11A01667 (HORIBA) kits.

### 2.3. Brain Sampling, mRNA Isolation and RT-qPCR

The hypothalamus obtained in experiment 1 was isolated by two frontal sections at the optic chiasm and the mammillary bodies. The hypothalamic region was isolated by two diagonal sections from the fornix to the most ventro-lateral site of the optic tract. The right hypothalamic hemisphere was transferred to a 4% Formafix solution (Grimm Logistik GmbH, Torgelow, Germany) and stored at 4 °C for at least 4 weeks. From the left hypothalamus, the paraventricular nucleus (PVN) and arcuate nucleus (ARC) were isolated, immediately snap frozen in liquid nitrogen and stored at −80 °C until further analysis. Briefly, tissue samples were ground under liquid nitrogen and total RNA was extracted from 19 to 43 mg tissue powder using an innuPREP RNA Mini Kit 2.0. Residual DNA was removed with an innuPREP DNase I Digest Kit (Analytik Jena AG, Jena, Germany). Integrity and the quality of the obtained mRNA was confirmed after electrophoresis on agarose gel stained with ethidium bromide (Carl Roth GmbH) and by measurement of the optical density 260:280 ratio. RNA concentrations were determined spectrophotometrically on a NanoPhotometer (Implen GmbH, Munich, Germany). Subsequently, 750 ng of total RNA was reverse-transcribed with a SensiFAST cDNA Synthesis Kit (Bioline, London, UK). Real-time quantitative PCR was performed on a LightCycler 2.0 (Roche, Basel, Switzerland) with 2 µL of cDNA using the SensiFAST SYBR No-ROX Kit (Bioline). Each cDNA sample was analyzed in duplicate. The efficiency of amplification was calculated using LinRegPCR software, version 2014.4 (Academic Medical Centre, Amsterdam, The Netherlands), yielding efficiency values between 1.81 and 1.92 (Table S2). Data were quantified using qbasePlus software (Biogazelle, Gent, Belgium) and normalized to the most stable genes, eukaryotic translation initiation factor 3 subunit K (*EIE3K*) and peptidylprolyl isomerase A (*PPIA*). Primers used for the analysis of orexigenes and genes related to the ECS are shown in Table S2. Due to the limited amount of mRNA obtained from the left ARC of some animals, expression analysis for *NPY* and *PLAAT5* could only be performed for NaCl (*n* = 7), AEA (*n* = 7), 2-AG (*n* = 4), and NaCl (*n* = 5), AEA (*n* = 6), 2-AG (*n* = 6), respectively.

### 2.4. Immunohistochemistry (IHC)

Tissue of the right hypothalamic hemisphere was dehydrated in a series of ethanol solutions of increasing concentrations up to 100%, embedded in paraffin, and subsequently cut on a microtome (Leica RM2145, Wetzlar, Germany) in 4 µm sections. Slices were mounted on Dako Flex IHC slides and dried in an incubator for one hour at 60 °C. For the analysis of CB_1_-positive cells, slices were pre-treated to block peroxidases as recently described [35]. Slices were incubated overnight with a polyclonal rabbit IgG against CB_1_ (Abcam, Cambridge, UK; ab23703; 1:500) followed by 1 h incubation with a goat anti-rabbit HRP antibody (Agilent Dako, Santa Clara, CA USA; 1:100) at room temperature. Immunoreactivity was visualized with 3,3′-diaminobenzidine (DAB) precipitation using the LSAB2 System-HRP kit (Dako; K0675). Slices were counterstained with hemalaun to visualize cell nuclei. Negative controls were incubated omitting the primary antibody. No unspecific binding of the secondary antibody was detected. Slices were visualized with a BX51 light microscope (OLYMPUS, Hamburg, Germany) equipped with 20× magnification and an SC50 camera (OLYMPUS) with the cellSens Standard software (OLYMPUS). Five to ten images of the PVN were taken per animal. For blinded image analysis, image colors were de-convoluted using the IHC profiler of the ImageJ software (National Institutes of Health, Bethesda, MD, USA) [39]. The pixel intensities of DAB images were analyzed using the color threshold method of ImageJ with brightness ranging from 229 to 245.

For immunofluorescence-based co-localization studies, slices were incubated with a polyclonal guinea pig anti-AgRP antibody (Abcam; ab228495, 1:200) together with a rabbit-anti-cFos antibody (Abcam; ab99515, 1:100). After overnight incubation, the slices were washed 3 times in phosphate-buffered saline (pH 7.4) for 10 min, followed by incubation with secondary fluorescence-labeled antibodies: goat-anti-guinea pig Alexa Fluor 488 (Thermo Fisher Scientific, Waltham, MA USA; 1:500) and goat-anti-rabbit DyLight 594 (Thermo Fisher Scientific; dilution 1:100) for 1 h at room temperature. The slices were counterstained with DAPI (AppliChem GmbH, Germany) for 15 min. For the negative control, slices were incubated omitting the primary antibody. No unspecific binding of the secondary antibody was detected. For the co-localization between Orexin A and cFos, primary sheep-anti-Orexin A antibody (NB100-65204; Bio-Techne, Minneapolis, MN, USA; 1:200) and secondary donkey-anti-sheep antibody (Alexa Fluor 488, Thermo Fisher Scientific; 1:100) were applied by using the same protocol as described above. Slices were analyzed with an Axioskop 40 FL fluorescence microscope (ZEISS, Oberkochen, Germany) at 20× magnification and images were acquired with an AxioCam MRc5 camera, controlled by ZEN 2 lite software (ZEISS). For AgRP analysis, four to five images per animal were taken and analyzed blinded using an automated quantitative analysis of ImageJ (NIH). Briefly, images were converted to 16 bit greyscale and the threshold was manually adjusted to only highlight and select the cell structures. After obtaining the binary image, pixels were counted according to the following criteria: size (pixel^^^2): 0.01-Infinity and circularity: 0.00–1.00. For Orexin A analysis, two to four images were taken per animal, but due to technical biases, three slices could not be evaluated, resulting in the analysis of NaCl (*n* = 6), AEA (*n* = 6) and 2-AG (*n* = 5). Image analysis was performed blinded using the Cell Counter plugin of the ImageJ software (NIH) for manual cell counting.

### 2.5. Statistical Analyses

Statistical analyses were performed using the SAS software for Windows, version 9.4 (SAS Institute Inc., Cary, NC, USA). Data were analyzed with repeated measurement analyses of variance using the MIXED procedure in SAS/STAT software. DMI/BW^0.75^ and cumulative DMI/BW^0.75^ were evaluated on day 8 in hourly intervals between 1 and 6 h post-injection. The respective data were statistically analyzed in hourly intervals with the fixed effects treatment (NaCl, AEA and 2-AG), diet (GS and CS) and time (interval), with diet and time as repeated variables. Plasma AEA and 2-AG concentrations were also analyzed using repeated measures ANOVA with fixed effects for treatment, diet and time of sampling, with diet and time as repeated variables. Data obtained from IHC and mRNA expression analyses were first analyzed using ANOVA; for mRNA expression analysis, the model contained the fixed effects treatment and diet, and for IHC analysis, the model contained the fixed effects treatment, diet and image, with image as the repeated variable. Because the effect of diet was found not significant, IHC and mRNA data were subsequently analyzed using ANOVA, including the fixed effect treatment (for mRNA) or the fixed effects treatment, and image, with image as the repeated variable (for IHC analysis). Data were tested for normal distribution using the Shapiro–Wilk test and transformed using Johnson transformation prior to testing. To evaluate the EC, NEFA and glucose concentrations in experiment 2, the model contained the fixed effect time, with time as the repeated variable. The calculation of Pearson correlation between NEFA, glucose and EC concentrations was performed by using the CORR procedure of SAS. Least square means (LSMs) and their standard error (SE) were calculated and pairwise tested for each fixed effect in the models described above by using the Tukey–Kramer procedure for pairwise multiple comparisons. Effects and differences at *p* < 0.05 were considered significant.

## 3. Results

### 3.1. Experiment 1

#### 3.1.1. Body Weight, Milk Yield and Intake

**Figure 1:**
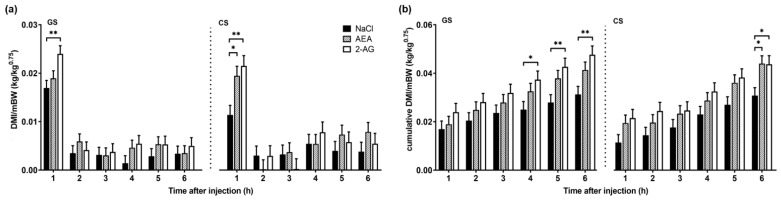
Dry matter intake per metabolic body weight DMI/BW^0.75^ (**a**) and cumulative DMI/BW^0.75^ (**b**) on day 8, 1 to 6 h after intraperitoneal administration with NaCl (*n* = 7), AEA (*n* = 7) or 2-AG (*n* = 6). Cows were fed ad libitum a grass silage (GS) and corn silage (CS)-based diet. Data are shown as LSM ± SE. Treatment differences are indicated by * *p* < 0.05 and ** *p* < 0.01 (Tukey–Kramer). ANOVA results are presented in Table S3.

Body weight, BW^0.75^ and milk yield did not differ between groups (data not shown). The DMI normalized to metabolic body weight (DMI/BW^0.75^) was assessed on day 8 for 6 h after administrations and was found to be different between the treatment groups (*F*(2,36) = 7.73, *p* < 0.01) and over time (*F*(6,106) = 73.3, *p* < 0.001) but not between diets (*F*(1,30) = 0.03, *p* = 0.87) (Figure 1a). More specifically, within the first h after injection, i.p. 2-AG administration resulted in a 41.5 and 88.8% higher DMI/BW^0.75^ compared to the control group (*p* < 0.01) on the GS and CS diet, respectively. During the same time, i.p. AEA compared to the control administration increased DMI/BW^0.75^ only by 11.8% on the GS diet, but by 70.7% on the CS diet (*p* < 0.05). Between 2 and 6 h post-treatment, DMI/BW^0.75^ was not significantly different between groups. Likewise, cumulative DMI/BW^0.75^ was different between treatment groups (*F*(2,28) = 4.10, *p* < 0.05) but not between diets (*F*(1,39) = 1.93, *p* = 0.17). Cumulative DMI/BW^0.75^ increased over time (*F*(6,88) = 221.09, *p* < 0.001), resulting in a 52.5 and 42.0% higher intake 6 h after i.p. 2-AG administration compared to the control group on the GS and CS diet, respectively (*p* < 0.05, Figure 1b). The i.p. AEA administration increased the 6 h cumulative DMI/BW^0.75^ by 32.2 and 42.8% compared to the control group on the GS and CS diet, respectively (*p* < 0.05). 

#### 3.1.2. Plasma Endocannabinoid Concentrations

**Figure 2:**
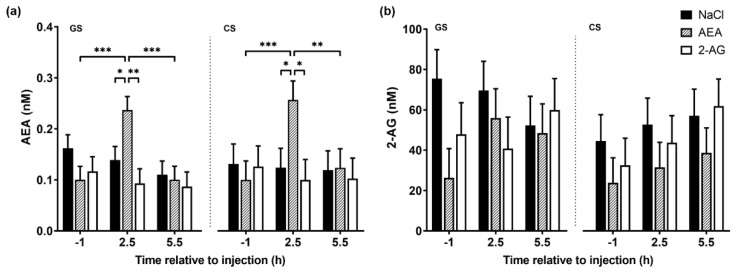
Plasma endocannabinoid concentrations on day 8, 1 h before, 2.5 h and 5.5 h after intraperitoneal administration with NaCl (*n* = 7), AEA (*n* = 7) or 2-AG (*n* = 6). Cows were fed ad libitum a grass silage (GS) and corn silage (CS)-based diet. Data for (**a**) AEA (nM) and (**b**) 2-AG (nM) are shown as LSM ± SE. Treatment and time differences are indicated by * *p* < 0.05, ** *p* < 0.01 and *** *p* < 0.001 (Tukey–Kramer). ANOVA results are presented in Table S3.

We next examined whether i.p. EC injection affected plasma AEA and 2-AG concentrations. Overall, plasma AEA concentrations were not different between treatment groups (*F*(2,29) = 0.96, *p* = 0.40). However, plasma AEA levels changed over time in the AEA-treated group (*F*(2,40) = 3.41, *p* < 0.05). Relative to the pre-injection level, the i.p. administration of 5 µg/kg AEA increased plasma AEA concentrations 2.5 h post-injection to 136.7 and 156.9% on the GS and CS diet, respectively (*p* < 0.001; Figure 2a). This increase was significantly higher compared to the control and 2-AG group on both diets (*p* < 0.05). From 2.5 to 5.5 h after AEA injection, plasma AEA concentrations declined to baseline levels on the GS (*p* < 0.001) and CS (*p* < 0.01) diet. By contrast, plasma AEA concentrations remained constant in the control and 2-AG group. Furthermore, there was no change in plasma 2-AG concentrations after AEA, 2-AG or NaCl treatments (*F*(2,27) = 1.14, *p* = 0.34) or over time (*F*(2,37) = 1.44, *p* = 0.25) (Figure 2b). The diets had no effect on either AEA or 2-AG levels (*F*(1,37) = 0.21, *p* = 0.65) (*F*(1,21) = 2.41, *p* = 0.14), respectively.

#### 3.1.3. Transcription of Hypothalamic Genes

Analysis of mRNA abundance of the orexigenic neuropeptides AgRP and NPY in the ARC tended to be different between treatment groups (*F*(2,17) = 3.13, *p* = 0.07) (*F*(2,15) = 2.81, *p* = 0.09), respectively (Table 1). More specifically, the abundance of *AGRP* was approximately 70% and the abundance of *NPY* 78% lower in AEA compared to the control and 2-AG group, respectively. However, these differences were not observed in the PVN. The mRNA abundances of genes involved in the ECS (*CB1*, *DAGLA*, *FAAH*, *NAPLD* and *PLAAT5*) of the ARC and PVN were not different between treatment groups (Table 1).

#### 3.1.4. Immunohistochemistry

**Figure 3:**
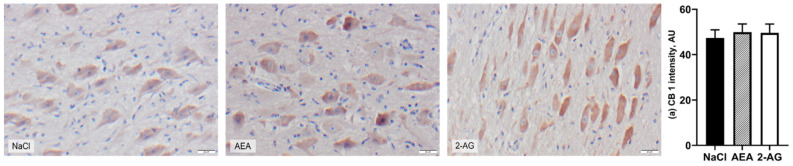
Representative images of DAB-stained CB1-expressing neurons in the periventricular nucleus (PVN) of cows after intraperitoneal administration with NaCl (*n* = 7), AEA (*n* = 7) or 2-AG (*n* = 6) for 9 days. CB1 immunoreactivity was visualized with DAB (arbitrary units, AU) and nuclei were stained with hemalaun (blue). Scale bar within images indicates 20 µm. CB1 intensities (**a**) are shown as LSM ± SE with *F*(2,15) = 0.15, *p* = 0.86 (ANOVA).

**Figure 4:**
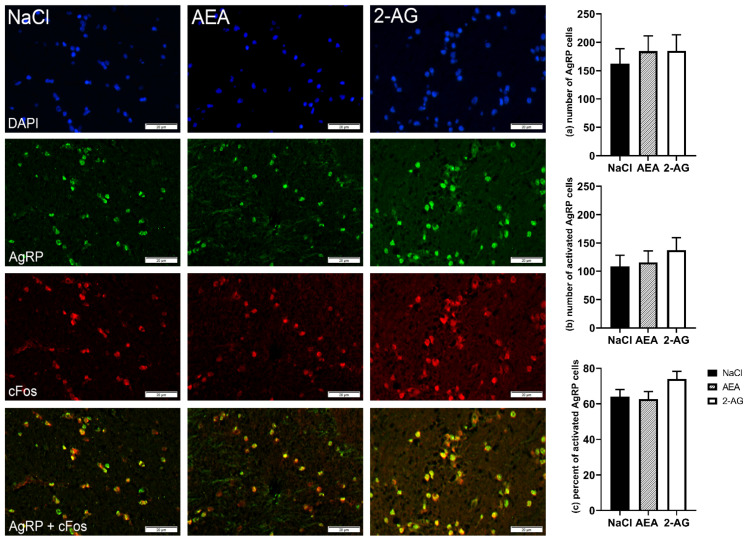
Immunofluorescence staining and representative images of the arcuate nucleus (ARC) of cows after intraperitoneal administration with NaCl (*n* = 7), AEA (*n* = 7) or 2-AG (*n* = 6) for 9 days. DAPI-stained nuclei (blue), AgRP neurons (green), cFos (red) and co-localization (merged image). Scale bar within images indicates 20 µm. Number of AgRP neurons (**a**), number of cFos-positive AgRPcells (**b**) and the percentage of activated AgRP cells per total AgRP cells (**c**). Data are shown as LSM ± SE with (**a**) *F*(2,17) = 0.25, *p* = 0.80, (**b**) *F*(2,17) = 0.51, *p* = 0.61, (**c**) *F*(2,15) = 2.11, *p* = 0.16 (ANOVA).

**Figure 5:**
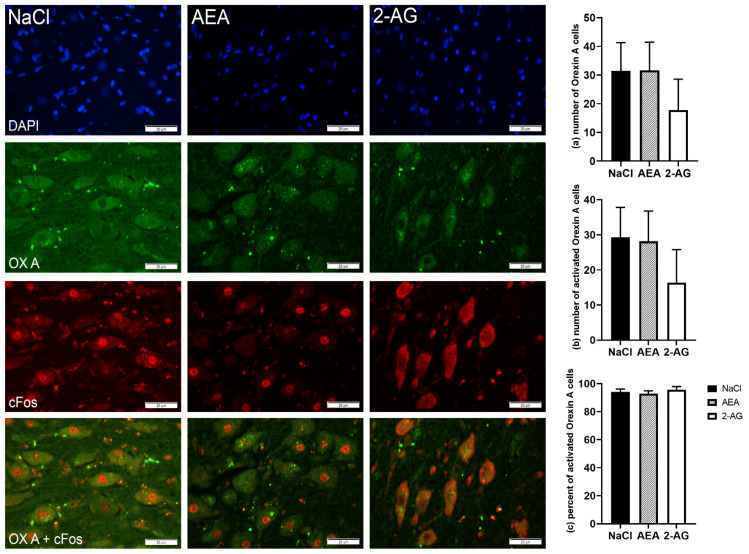
Immunofluorescence staining and representative images of the lateral hypothalamus (LH) of cows after intraperitoneal administration with NaCl (*n* = 6), AEA (*n* = 6) or 2-AG (*n* = 5) for 9 days. DAPI-stained nuclei (blue), orexin-A neurons (green), cFos (red) and co-localization (merged image). Scale bar within images indicates 20 µm. Number of orexin-A neurons (**a**), number of cFos-positive orexin-A cells (**b**) and the percentage of activated orexin-A cells per total orexin-A cells (**c**). Data are shown as LSM ± SE with (**a**) *F*(2,14) = 0.58, *p* = 0.57, (**b**) *F*(2,14) = 0.61, *p* = 0.56, (**c**) *F*(2,13) = 0.45, *p* = 0.65 (ANOVA).

When analyzed on the protein level, CB_1_ immunoreactivity of neurons located in the PVN was not different between groups (*F*(2,15) = 0.15, *p* = 0.86) (Figure 3). Despite the strong immunoreactivity of AgRP neurons in the ARC, both the number of AgRP cells (*F*(2,17) = 0.25, *p* = 0.80) and the number of cFos-positive AgRP cells (*F*(2,17) = 0.51, *p* = 0.61) were not statistically different between treatment groups (Figure 4). In addition, the resulting percentage of activated AgRP cells per total number of AgRP cells was also not different (*F*(2,15) = 2.11, *p* = 0.16). Likewise, there were no differences between treatment groups for orexin-A (*F*(2,14) = 0.58, *p* = 0.57), cFos immunofluorescence staining (*F*(2,14) = 0.61, *p* = 0.56) and the resulting percentage of activated orexin-A cells per total number of orexin-A cells in the LH (*F*(2,13) = 0.45, *p* = 0.65) (Figure 5).

### 3.2. Experiment 2

#### Food Deprivation

Plasma 2-AG concentrations increased 7 h after food withdrawal relative to the basal level (*p* < 0.05, Table 2). This increase was accompanied by increased plasma NEFA (*p* < 0.01) and decreased plasma glucose concentrations (*p* < 0.01). After refeeding, plasma 2-AG (*p* < 0.05), but not plasma NEFA and glucose concentrations, returned to baseline levels. Pearson correlation analysis including samples from all time points investigated reveal that 2-AG and NEFA concentrations were positively correlated (*r* = 0.63; *p* < 0.01) and 2-AG and glucose concentrations were negatively correlated (*r* = −0.50; *p* < 0.05). In addition, plasma AEA concentrations did not change in response to food deprivation.

## 4. Discussion

### 4.1. Effect of Endocannabinoids on Food Intake

The present study demonstrates that systemically administered 2-AG and AEA increase food intake in cows in the short term. Specifically, the i.p. administration of 2.5 µg 2-AG/kg BW (on GS and CS) as well as 5 µg AEA/kg BW (on CS) resulted in a higher increase in food intake during the first hour after injection compared to the control group. Although the differences in food intake were not apparent when analyzed for the subsequent hourly intervals, cumulative food intake of 2-AG animals was greater 4 to 6 h on the GS, and of AEA-treated cows 6 h post-injection on the CS diet. Despite repeated daily i.p. administrations, food intake of cows did not increase in the long term, e.g., over 8 days [40]. In contrast, daily i.p. administrations of 1 µg AEA and 2-AG per kg BW increased food intake in mice over 7 and 14 days [20,22]. However, the administration of a single EC dose into the brain of mice only increased food intake for 1 h post-injection [41]. The observed short-term effect of EC on food intake might be due to the short half-life of AEA and 2-AG lasting only for a few minutes in mice [17,18].

The EC-mediated control of food intake is complex, and CB_1_ activation has been shown to promote food intake at various levels in the CNS [42]. For example, peripheral [43] and central [44] CB_1_ activation influences the motivation to consume palatable foods and promotes food intake through increased odor detection in the main olfactory bulb of mice [45]. Although our study did not aim to investigate the interaction between ECs and palatability, it is tempting to speculate that stronger impact of AEA and 2-AG on the CS compared to GS intake is due to the higher increase in palatability of the CS diet. Because the CS and GS rations did not have the same energy content, it may also be the case that AEA and 2-AG administrations particularly support the ingestion of high energy density diets. Therefore, it would be of great interest to investigate the effect of ECs on taste and energy content preferences as wells as hedonic aspects of food intake in ruminants in the near future.

### 4.2. Effect of Endocannabinoids on Plasma AEA and 2-AG Concentration

In addition to the direct effect on splanchnic EC receptors and vagal afferent neurons [10,12,13], i.p.-administered ECs can be rapidly (seconds to minutes) absorbed from the peritoneal cavity into systemic circulation due to their low molecular weight and high lipophilicity [46]. The high lipophilicity allows EC to be rapidly removed from the circulation and distributed into tissues. Willoughby et al. [17] demonstrated that radiolabeled AEA (50 mg/kg) was detectable in the brain of mice as early as 1 min after i.v. injection. In our study, we did not sample plasma before 2.5 h post-administration and thus may have missed the time of highest accumulation of ECs in the circulation. However, the time point of plasma sampling 2.5 h post-administration was chosen to coincide with the collection of hypothalamic tissue. Plasma AEA concentrations were significantly increased 2.5 h post-injection of AEA, whereas AEA concentrations remained stable after 2-AG or NaCl administration. This finding suggests that i.p.-administered AEA accumulates in the circulation of cows and has a longer half-life than only a few minutes, as found in rodents [17]. Furthermore, Kozak et al. [47] demonstrated a much longer half-life of 2-AG when incubated with human plasma as compared to rat plasma, suggesting that the rat is not a suitable model to study the biological activities of ECs in humans. Our findings imply a longer half-life, at least for AEA, but whether the cow provides a more adequate model requires further investigation in the future. Moreover, our findings are in agreement with the results from Oddi et al. [15], demonstrating that AEA is transiently stored in lipid droplets in adipose tissue and can potentially re-enter the circulation with a timely delay. Previous studies also showed a biphasic pattern in radioactivity enrichment in plasma and various tissues after the i.v. administration of tracer-labeled AEA in mice [17], further supporting the idea of a longer-lasting exchange of AEA between the circulation and tissues. However, the bioavailability of AEA distinguishes from the bioavailability of 2-AG, because we did not observe changes in plasma 2-AG concentrations 2.5 or 5.5 h after i.p. 2-AG administration. Either 2-AG is stored longer in tissues or, more likely, has a shorter half-life than AEA. A tracer study with higher frequency sampling would be needed to determine the exact half-life for AEA and 2-AG.

### 4.3. Effect of Endocannabinoids on Hypothalamic Orexigenic Circuits

By binding to CB_1_ receptors expressed by vagal afferent neurons [10,12,13,48] and rapidly crossing the blood–brain barrier [49], peripherally administered ECs can modulate the release and action of neurotransmitters [19]. To elucidate these neuromodulatory effects, for the first time, we investigated how peripherally administered AEA and 2-AG affect hypothalamic orexigenic neuropeptides and genes related to the ECS. Central CB_1_ receptors play an essential role in the neuronal network of food intake and appetite stimulation [42]. In fact, Soria-Gómez et al. [50] demonstrated that AEA administration into the nucleus accumbens shell (NAcS) of rats did not only increase food intake but also cFos immunoreactivity in hypothalamic nuclei. Furthermore, presynaptic CB_1_ activation led to a suppressed inhibitory tone in perifornical LH neurons [51]. By using 3D reconstruction of serial ARC sections of mice, Morozov et al. [26] demonstrated that CB_1_-positive synapses innervate NPY/AgRP neurons, further strengthening an EC-mediated retrograde disinhibition of these orexigenic neurons. Moreover, in rat hypothalamic explants, a direct effect of ECs on hypothalamic NPY has been shown [25]. Specifically, the pharmacological stimulation of CB_1_ receptors with AEA and the synthetic agonist CP55940 increased hypothalamic NPY release. Based on these findings, we expected the activation of hypothalamic AgRP and NPY after enhancing the systemic EC tone. Conversely, AgRP immunoreactivity was not affected by either treatment, and *AGRP* and *NPY* mRNA abundances in the ARC of AEA-administered animals tended to be markedly lower than in the 2-AG and control groups. In addition, the expression of genes related to ECS and CB_1_ immunoreactivity were not affected by i.p. AEA and 2-AG injection. Failure to detect increased CB_1_ expression or activation of NPY/AgRP neurons might be due to time between i.p. injection and tissue gain of 2 to 3 h. Although AEA concentrations in the circulation were increased after 2.5 h, food intake did not differ in the second and third hour after AEA administration. This finding suggests that the activation of orexigenic neurons occurred presumably in the first hour after EC administration and could not be detected after 2 to 3 h. A collection of tissue samples 1 h after injection and during feeding would have been preferable; however, due to the necessary intermediary transport of the animals to the institute’s slaughterhouse, this could not be achieved. Another reason might have been an increased stress level during the transport of the animals to the slaughterhouse, which may have suppressed the former EC-mediated activation of NPY/AgRP neurons. Nonetheless, NPY and AgRP mRNA expression in the ARC of AEA-treated cows was lower than in control animals, which could be a counter-regulatory mechanism reducing the preceded activation of NPY/AgRP neurons. The direct interaction between ECs and the OX system has been demonstrated in several studies, i.e., by the co-expression of CB_1_ and OX receptors in various cell lines [28,29,52]. Furthermore, EC-mediated CB_1_ activation enhanced OX-A release by reducing the inhibition of respective neurons [27], and the inverse CB_1_ agonist AM251 decreased the expression of OX-A in the hypothalamus of rats [53]. In our study, we did not detect an increase in OX-A immunoreactivity or the c-FOS activation of OX-A-expressing neurons despite coinciding increased circulatory AEA concentrations. Again, food intake was not different between treatment groups in the second and third hour after EC administration, and thus we can only speculate whether OX-A-expressing neurons were activated in the first hour after AEA administration when food intake was higher than in control animals or if transport stress to the slaughterhouse suppressed EC-induced OX-A activation. Another important aspect is that ECs interact with anorexic circuits. For example, CB_1_ activation mediates the downregulation of POMC synthesis and α-MSH release in the ARC of mice [54]. In the present study, we did not analyze the expression of anorexic neuropeptides, which, however, should be considered in future studies to better understand the EC-mediated regulation of food intake in the bovine brain. Furthermore, EC tone in the hypothalamus could not be analyzed herein because the brain was contaminated with blood after captive bolt stunning.

### 4.4. Effect of Food Deprivation on Plasma Endocannabinoid Concentrations

A series of studies have investigated the effect of food deprivation on EC tone in the brain and small intestine of rodents [21,41,55]. Specifically, fasting resulted in increased 2-AG levels in the limbic forebrain, hypothalamus, and jejunal mucosa of rats [41,55]. Furthermore, fasting increased AEA levels in the small intestine of rats [21] and the blood of healthy, normoweight humans [56,57].

In the current study, plasma 2-AG significantly increased after 7 h of food deprivation and decreased to baseline levels after refeeding, whereas plasma AEA remained unchanged. The latter findings are consistent with the findings of Kirkham et al. [41], indicating that, as in rats, 2-AG in cows is most sensitive to short-term fasting and may be more involved in the immediate response to changes in energy supply by inducing appetite. Indeed, increased plasma 2-AG levels could originate from an increased 2-AG production in the small intestine [21,55] or from visceral adipose tissue [58]. However, our results are in contrast with findings in humans, where AEA levels increased with fasting [57], but no meal-related changes were observed for 2-AG [59]. Whether this indicates an inter species difference or more likely is due to time-dependent variations of EC levels in response to energy deficits [60] needs to be determined in future studies.

To confirm that the 7 h of food withdrawal caused an energy deficit, we analyzed plasma NEFA and glucose concentrations. Plasma NEFA concentration has been shown to serve as a reliable marker of fat mobilization in cows [61]. Increased plasma NEFA concentrations after 7 h of food withdrawal indicate a negative energy balance and were positively correlated with plasma 2-AG concentrations, whereas plasma glucose concentrations were inversely correlated with 2-AG. In support of the latter finding, a negative correlation for 2-AG with fasting glucose was also reported in humans [62]. However, an earlier study in cows showed a positive correlation between plasma AEA and plasma NEFA, but not between 2-AG and NEFA [35]. These variabilities call for further research to better understand the interaction of EC and energy metabolites.

## 5. Conclusions

The present pilot study highlights the cow as an animal model for endocannabinoid research and demonstrates that low doses of systemically administered 2-AG and AEA increase food intake in the short-term. AEA injections increased plasma AEA levels within 2.5 h after administration, indicating that i.p.-administered AEA accumulates in the circulation of cows. This finding indicates a longer half-life than only a few minutes found in mice, demonstrating the advantage of using a cow as an animal model for endocannabinoid research. However, the bioavailabilities of AEA and 2-AG are different. Despite higher plasma AEA concentrations, hypothalamic CB_1_, AgRP, and OX-A immunoreactivity was not different between AEA and control animals, suggesting that the activation of orexigenic neurons occurred presumably in the first hour after EC administration, as seen by the differences in food intake between groups at this time. Therefore, future studies should focus on the investigation of hypothalamic orexigenes already 1 h after i.p. EC administration. Nevertheless, downregulated NPY and AgRP mRNA expression in the ARC of AEA-treated animals indicate a counter-regulatory mechanism reducing the preceded activation of NPY/AgRP neurons. In addition, short-term food deprivation increased plasma 2-AG, while plasma AEA remained unchanged, suggesting that 2-AG is more sensitive to fasting than AEA in cows and may be more involved in the immediate response to changes in energy supply.

## Figures and Tables

**Table 1:** Relative mRNA abundances (LSM ± SE) of orexigenic neuropeptides and genes related to the ECS of the arcuate nucleus (ARC) and paraventricular nucleus (PVN). Samples were obtained from cows treated with NaCl (*n* = 7), AEA (*n* = 7) or 2-AG (*n* = 6) for 9 days.

Gene	NaCl	AEA	2-AG	ANOVA
ARC				
*AGRP*	1.72 ± 0.46	0.55 ± 0.04	2.03 ± 0.87	*F*(2,17) = 3.13, *p* = 0.07
*CNR1*	1.03 ± 0.16	1.06 ± 0.17	1.11 ± 0.14	*F*(2,17) = 0.06, *p* = 0.95
*DAGLA*	1.00 ± 0.09	0.98 ± 0.09	1.07 ± 0.07	*F*(2,17) = 0.29, *p* = 0.75
*FAAH*	1.07 ± 0.18	0.98 ± 0.10	1.12 ± 0.07	*F*(2,17) = 0.31, *p* = 0.73
*NAPELD*	1.05 ± 0.03	0.95 ± 0.11	1.07 ± 0.11	*F*(2,17) = 0.53, *p* = 0.60
NPY *	1.84 ± 0.70	0.42 ± 0.11	1.91 ± 0.48	*F*(2,15) = 2.81, *p* = 0.09
PLAAT5 *	1.16 ± 0.45	1.43 ± 0.42	1.05 ± 0.23	*F*(2,14) = 0.39, *p* = 0.68
PVN				
*AGRP*	1.17 ± 0.17	1.15 ± 0.38	1.48 ± 0.47	*F*(2,17) = 0.24, *p* = 0.79
*CNR1*	0.91 ± 0.07	1.07 ± 0.24	1.37 ± 0.24	*F*(2,17) = 1.12, *p* = 0.35
*DAGLA*	0.99 ± 0.06	1.06 ± 0.10	1.00 ± 0.14	*F*(2,17) = 0.16, *p* = 0.85
*FAAH*	0.99 ± 0.10	1.00 ± 0.07	1.08 ± 0.09	*F*(2,17) = 0.29, *p* = 0.75
*NAPELD*	1.01 ± 0.09	1.06 ± 0.09	1.00 ± 0.08	*F*(2,17) = 0.12, *p* = 0.89
*PLAAT5*	1.38 ± 0.24	1.26 ± 0.46	1.65 ± 0.66	*F*(2,17) = 0.18, *p* = 0.84

* Because of the limited amount of ARC mRNA, evaluable results were limited to NaCl (*n* = 7), AEA (*n* = 7), 2-AG (*n* = 4) and NaCl (*n* = 5), AEA (*n* = 6), 2-AG (*n* = 6), respectively. AGRP, agouti-related neuropeptide; CNR1, cannabinoid receptor 1; DAGLA, diacylglycerol lipase alpha; FAAH, fatty acid amide hydrolase; NAPEPLD, N-acyl phosphatidylethanolamine phospholipase D; NPY, neuropeptide Y; PLAAT5, phospholipase A and acyltransferase 5.

**Table 2:** Plasma endocannabinoid (AEA, 2-AG), NEFA and glucose concentrations of 7 cows with ad libitum intake, after 7 h of food deprivation and 17 h after refeeding. Cows were offered a corn silage-based diet. Data are shown as LSM + SE.

	Ad Libitum	Food Deprivation	Refeeding	ANOVA
AEA (nM)	0.26 ^a^ ± 0.02	0.29 ^a^ ± 0.02	0.27 ^a^ ± 0.02	*F*(2,12) = 0.69, *p* = 0.52
2-AG (nM)	3.60 ^a^ ± 0.49	4.73 ^b^ ± 0.49	3.63 ^a^ ± 0.49	*F*(2,12) = 6.17, *p* < 0.05
NEFA (µmol/L)	162.71 ^a^ ± 40.28	237.14 ^b^ ± 40.28	189.29 ^ab^ ± 40.28	*F*(2,12) = 7.46, *p* < 0.01
Glucose (mmol/L)	4.06 ^a^ ± 0.13	3.65 ^b^ ± 0.13	3.73 ^b^ ± 0.13	*F*(2,12) = 8.38, *p* < 0.01

^a,b^ Different superscript letters within one row indicate significance differences (*p* < 0.05, Tukey–Kramer).

## Data Availability

All data generated and analyzed are available from the corresponding author on request.

## Supplementary Materials

The following are available online at https://www.mdpi.com/article/10.3390/nu13103587/s1, Table S1: ingredients and chemical composition of diets, Table S2: primer sequences, Table S3: *p*-values.
