# Supplementary material for: A Role for Peripheral Anandamide and 2-Arachidonoylglycerol in Short-Term Food Intake and Orexigenic Hypothalamic Responses in a Species with Continuous Nutrient Delivery
Source: Nutrients. 2021 Oct 13;13(10):3587. doi: 10.3390/nu13103587 (PMC8540326; doi:10.3390/nu13103587)
Supplement: Supplementary file 1 [file nutrients-13-03587-s001.zip › nutrients-1367530-supplementary.pdf]

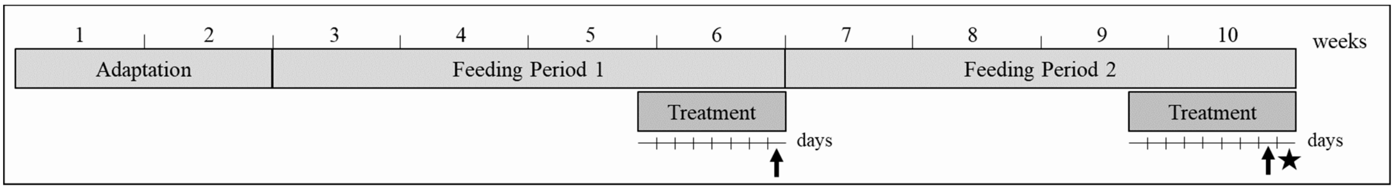


**Figure S1.** Study design, experiment 1. Animals were fed a grass silage (GS) and corn silage (CS) based diet (ad libitum) in a cross over design in feeding period 1 and 2. Animals received the same treatment in each period, either NaCl (n = 7), AEA (n = 7) or 2-AG (n = 6). Arrow indicates blood samples taken on day 8 of each treatment period. Star indicates end point analysis of brain tissue.

**Table S1.** Ingredients and chemical composition (means ± SE) of the grass silage (GS) and corn silage (CS) based total mixed ration (TMR) of Experiment 1 and 2.

| Component | Experiment 1 | | Experiment 2 |
|---|---|---|---|
| | GS | CS | CS |
| Ingredient, g/kg of DM | | | |
| Grass silage | 464.9 ± 18.1 | | |
| Corn silage | | 570.9 ± 7.3 | 558.3 |
| Barley straw | | 57.7 ± 13.9 | 85.4 |
| Grass hey | 131.9 ± 17.1 | | |
| Corn, ground | 114.9 ± 18.9 | 78.7 ± 11.0 | 56.8 |
| Wheat, ground | 116.5 ± 1.5 | 44.4 ± 0.5 | 43.6 |
| Extracted soy meal | | 95.8 ± 5.2 | 106.0 |
| Extracted rapeseed meal | | 134.8 ± 1.7 | 132.5 |
| Concentrate[1] | 114.4 ± 5.1 | | |
| Mineral/vitamin mix[2] | 7.4 ± 0.1 | 9.9 ± 0.1 | 9.7 |
| Lime stone powder[3] | | 5.1 ± 0.1 | 5.0 |
| Animal feed salt[4] | | 2.7 ± 0.0 | 2.7 |
| Chemical analysis, g/kg of DM | | | |
| Crude ash | 76.4 ± 2.1 | 40.6 ± 2.3 | 37.0 ± 4.0 |
| Crude protein | 161.6 ± 3.9 | 158.0 ± 3.5 | 159.5 ± 9.5 |
| Crude fiber | 190.8 ± 7.9 | 164.0 ± 2.3 | 161.5 ± 4.5 |
| Crude fat | 28.0 ± 1.4 | 34.0 ± 0.7 | 35.5 ± 0.5 |
| Starch | 184.2 ± 20.4 | 298.0 ± 7.3 | 315.5 ± 1.5 |
| aNDFom | 471.4 ± 17.3 | 385.4 ± 5.3 | 378.5 ± 13.5 |
| ADFom | 235.2 ± 11.1 | 198.8 ± 3.5 | 191.5 ± 5.5 |
| ME (MJ/kg of DM)[5] | 10.7 ± 0.2 | 11.5 ± 0.1 | 11.6 ± 0.1 |
| $NE_L$ (MJ/kg of DM)[5] | 6.5 ± 0.1 | 7.0 ± 0.1 | 7.1 ± 0.1 |

[1]MF 2000 (Ceravis AG, Regensburg, Germany): 30% extracted soy meal from peeled and steam-heated beans, 25.4% corn grit, 15% malt culms, 6.9% extracted rapeseed meal, 5.1% wheat, 5% sugar beet pulp, 4.9% dried grain pulp, 4.1% beet molasses, 2% $NaHCO_3$, 1% $CaCO_3$, 0.2% NaCl. Additives per kg original substance: 10,000 IU vitamin A, 1125 IU vitamin $D_3$, 40 mg vitamin E, 0.6 mg I , 0.4 mg Co , 50 mg Mn, 75 mg Zn, 0.4 mg Se. Composition: 24% CP, 2.6% crude fat, 5.1% crude fiber, 8% crude ash, 0.73% Ca, 0.5% P, 0.65% Na, 7.1 MJ NEL/kg.

[2]Panto®-Mineral R 8609 (HL Hamburger Leistungsfutter GmbH, Hamburg, Germany): 32.8% $CaNaPO_3$, 19.3% CaMg $(CO_3)_2$, 16.7% NaCl, 15.3% $CaCO_3$, 6.1% MgO, 3% sugar cane molasses.

Additives per kg original substance: 900,000 IU vitamin A, 200,000 IU vitamin $D_3$, 4.5 g vitamin E, 1.5 g Cu, 8 g Zn, 5 g Mn, 60 mg I, 70 mg Co, 50 mg Se. Composition: 20% Ca, 6% P, 8% Na, 6% Mg.

[3]Kreidekalk (Spezialfutter Neuruppin GmbH und Co.KG): $CaCO_3$. Composition: 37% Ca.

[4]ESCO Viehsalz (esco - european salt company GmbH, Hannover, Germany) Composition: 99% NaCl, 0.3% Ca, 0.01% Mg, <2 mg/kg Cu, < 2 mg/kg Zn

[5]ME and NEL content of TMR were calculated according to the recommendations of the German Society of Nutrition Physiology (GfE).

**Table S2.** Primer sequences

| Gene | | Primer sequence (5′ to 3′) | Accession no. | bp[1] | Efficiency |
|---|---|---|---|---|---|
| AGRP | fwd | ACCGTCGCTGAAGAGGATAA | NM_173983.2 | 214 | 1.87[a] |
| | rev | GCAGAAGGCGTTGAAGAAAC | | | 1.84[p] |
| CNR1 | fwd | AAGCCCG-CATGGACATTCGGCTGG | NM_001242341.2 | 79 | 1.91[a] |
| | rev | AGCAGAGGGCCCCAGCAGAT | | | 1.86[p] |
| DAGLA | fwd | GGCTCAAAGTGTTCCTCTGC | NM_001192583.3 | 107 | 1.85[a] |
| | rev | CACAATGTCCAGGTCTCGGA | | | 1.86[p] |
| EIE3K | fwd | CCAGGCCCACCAAGAAGAA | NM_001034489 | 125 | 1.85[a] |
| | rev | TTATACCTTCCAGGAGGTCCATGT | | | 1.81[p] |
| FAAH | fwd | TTCCTGCCAAGCAACATACCT | NM_001099102.2 | 105 | 1.85[a] |
| | rev | CACGAAATCACCTTT-GAAGTTCTG | | | 1.89[p] |
| MGLL | fwd | GCAACCAGCTGCTCAACAC | NM_001206681.1 | 154 | -- |
| | rev | AGCGTCTTGTCCTGGCTCTT | | | -- |
| NAAA | fwd | CAGCACTACGACCGGGACTT | NM_001100369 | 100 | -- |
| | rev | CCGGGACGACTTTTCTGATC | | | -- |
| NAPEPLD | fwd | AGAGATCACAGCAGCGTTCCAT | NM_001015680.1 | 95 | 1.92[a] |
| | rev | ACTCCAGCTTCTTCAGGGTCATC | | | 1.92[p] |
| NPY | fwd | GTACCCCTCCAAGCCTGAC | NM_001014845.3 | 191 | 1.84[a] |
| | rev | CCAGCCTAGTTCTGGGAATG | | | -- |
| PLAAT5 | fwd | AAGATCATCCAGCGGACAAAA | NM_001281908.2 | 100 | 1.86[a] |
| | rev | CGCCGTATCTCAGGTCATTGA | | | 1.86[p] |

| | | | | | |
|---|---|---|---|---|---|
| PLA2G4E | fwd | AGGGCCTTTGTCTCCAAGTT | XM_024998182 | 170 | -- |
| | rev | ACAAAGAATGCGGTGTCCAG | | | -- |
| PPIA | fwd | GGATTTATGTGCCAGGGTGGTGA | NM_178320 | 120 | 1.92[a] |
| | rev | CAAGATGCCAGGACCTGTATG | | | 1.89[p] |

[1] Product size (base pairs)

[a] Arcuate nucleus (ARC), [p] Paraventricular nucleus (PVN)

-- Due to insufficient amount of amplicon products ($C_t > 30$), the efficiency could not be calculated.

AGRP, agouti related neuropeptide; CNR1, cannabinoid receptor 1; DAGLA, diacylglycerol lipase alpha; EIE3K, eukaryotic translation initiation factor 3 subunit K; FAAH, fatty acid amide hydrolase; MGLL, monoglyceride lipase; NAAA, N-acylethanolamine acid amidase; NAPEPLD, N-acyl phosphatidylethanolamine phospholipase D; NPY, neuropeptide Y; PLA2G4E, phospholipase A2 group IVE; PLAAT5, phospholipase A and acyltransferase 5; PPIA, Peptidylprolyl isomerase A

**Table S3.** ANOVA of the effects of treatment, diet, housing and their interactions using the MIXED procedure in SAS (SAS Institute, Cary, NC, USA)

| | Treatment | Diet | Time | Treatment x diet | Treatment x time | Diet x time | Treatment x diet x time |
|---|---|---|---|---|---|---|---|
| Fig. 1A (DMI/BW$^{0.75}$ (kg/kg$^{0.75}$)) | $F(2,36) = 7.73$ $P < 0.01$ | $F(1,30) = 0.03$ $P = 0.87$ | $F(6,106) = 73.3$ $P < 0.001$ | $F(2,30) = 0.26$ $P = 0.78$ | $F(12,106) = 1.85$ $P < 0.05$ | $F(6,113) = 1.50$ $P = 0.18$ | $F(12,113) = 0.65$ $P = 0.80$ |
| Fig. 1B (cumulative DMI/BW$^{0.75}$ (kg/kg$^{0.75}$)) | $F(2,28) = 4.10$ $P < 0.05$ | $F(1,39) = 1.93$ $P = 0.17$ | $F(6,88) = 221.09$ $P < 0.001$ | $F(2,39) = 0.08$ $P = 0.92$ | $F(12,88) = 2.83$ $P < 0.01$ | $F(6,136) = 2.53$ $P < 0.05$ | $F(12,136) = 0.64$ $P = 0.80$ |

| | Treatment | Diet | Time | Treatment x diet | Treatment x time | Diet x time | Treatment x diet x time |
|---|---|---|---|---|---|---|---|
| Fig. 2A (Plasma AEA (nM)) | $F(2,29) = 0.96$ $P = 0.40$ | $F(1,37) = 0.21$ $P = 0.65$ | $F(2,40) = 3.41$ $P < 0.05$ | $F(2,37) = 0.82$ $P = 0.45$ | $F(4,40) = 4.29$ $P < 0.01$ | $F(2,75) = 1.52$ $P = 0.23$ | $F(4,75) = 0.33$ $P = 0.86$ |
| Fig. 2B (Plasma 2-AG (nM)) | $F(2,27) = 1.14$ $P = 0.34$ | $F(1,21) = 2.41$ $P = 0.14$ | $F(2,37) = 1.44$ $P = 0.25$ | $F(2,21) = 0.25$ $P = 0.78$ | $F(4,37) = 1.15$ $P = 0.35$ | $F(2,44) = 2.32$ $P = 0.11$ | $F(4,44) = 2.26$ $P = 0.08$ |
